# High frequency of BRCA1, but not CHEK2 or NBS1 (NBN), founder mutations in Russian ovarian cancer patients

**DOI:** 10.1186/1897-4287-7-5

**Published:** 2009-02-25

**Authors:** Evgeny N Suspitsin, Nathalia Yu Sherina, Daria N Ponomariova, Anna P Sokolenko, Aglaya G Iyevleva, Tatyana V Gorodnova, Olga A Zaitseva, Olga S Yatsuk, Alexandr V Togo, Nathalia N Tkachenko, Grigory A Shiyanov, Oksana S Lobeiko, Nadezhda Yu Krylova, Dmitry E Matsko, Sergey Ya Maximov, Adel F Urmancheyeva, Nathalia V Porhanova, Evgeny N Imyanitov

**Affiliations:** 1 Laboratory of Molecular Oncology, N.N. Petrov Institute of Oncology, St. Petersburg, Russia; 2 Department of Gynecology, N.N. Petrov Institute of Oncology, St. Petersburg, Russia; 3 Laboratory of Tumor Morphology, N.N. Petrov Institute of Oncology, St. Petersburg, Russia; 4 Department of Genetics, St. Petersburg Pediatric Medical Academy; Department of Oncology, St. Petersburg, Russia; 5 Department of Oncology, St. Petersburg Medical Academy of Postgraduate Studies, St. Petersburg, Russia; 6 Department of Gynecology, Regional Oncological Hospital, Krasnodar, Russia

## Abstract

**Background:**

A significant portion of ovarian cancer (OC) cases is caused by germ-line mutations in BRCA1 or BRCA2 genes. BRCA testing is cheap in populations with founder effect and therefore recommended for all patients with OC diagnosis. Recurrent mutations constitute the vast majority of BRCA defects in Russia, however their impact in OC morbidity has not been yet systematically studied. Furthermore, Russian population is characterized by a relatively high frequency of CHEK2 and NBS1 (NBN) heterozygotes, but it remains unclear whether these two genes contribute to the OC risk.

**Methods:**

The study included 354 OC patients from 2 distinct, geographically remote regions (290 from North-Western Russia (St.-Petersburg) and 64 from the south of the country (Krasnodar)). DNA samples were tested by allele-specific PCR for the presence of 8 founder mutations (BRCA1 5382insC, BRCA1 4153delA, BRCA1 185delAG, BRCA1 300T>G, BRCA2 6174delT, CHEK2 1100delC, CHEK2 IVS2+1G>A, NBS1 657del5). In addition, literature data on the occurrence of BRCA1, BRCA2, CHEK2 and NBS1 mutations in non-selected ovarian cancer patients were reviewed.

**Results:**

BRCA1 5382insC allele was detected in 28/290 (9.7%) OC cases from the North-West and 11/64 (17.2%) OC patients from the South of Russia. In addition, 4 BRCA1 185delAG, 2 BRCA1 4153delA, 1 BRCA2 6174delT, 2 CHEK2 1100delC and 1 NBS1 657del5 mutation were detected. 1 patient from Krasnodar was heterozygous for both BRCA1 5382insC and NBS1 657del5 variants.

**Conclusion:**

Founder BRCA1 mutations, especially BRCA1 5382insC variant, are responsible for substantial share of OC morbidity in Russia, therefore DNA testing has to be considered for every OC patient of Russian origin. Taken together with literature data, this study does not support the contribution of CHEK2 in OC risk, while the role of NBS1 heterozygosity may require further clarification.

## Background

Ovarian cancer (OC) is a major cause of oncological mortality in females, with a life-time risk approaching to approximately 1.7%. Poor outcome of OC is largely attributed to the failure to diagnose the disease at early, potentially curable stages; small tumors of the ovary are usually asymptomatic and likely to be missed by routine gynecological examination [1]. Genetic component may play a key role in OC etiology: depending on the country and ethnicity, 5–50% OC cases are attributed to the germ-line heterozygous inactivation of BRCA1 or BRCA2 genes (see additional file 1). Interestingly, while BRCA-related breast carcinomas (BC) are usually strongly enriched by early onset and familial cancer cases, this relationship is less evident for OC. Although the majority of the studies confirm some association between the presence of BRCA mutation and younger patients age (for BRCA1 but not BRCA2) or family history of the disease (see additional file 1), the strength of this trend is not enough to limit BRCA testing by particular categories of OC patients; instead, the OC diagnosis itself is often considered as a sufficient indication for BRCA analysis. Actually, the occurrence of BRCA mutations in randomly recruited OC cases is fairly similar to the estimates, which are obtained in preselected high-risk categories of women with breast cancer [2-41].

In Russia, a significant fraction of early-onset and/or bilateral and/or familial BC cases is caused by founder mutations in cancer genes [42]. Presence of the "founder effect" significantly decreases the costs of the DNA testing, thus relaxing the criteria for patients selection. While distribution of germ-line mutations in Russian breast cancer cases has been studied with sufficient level of comprehension, no systematic analysis has been undertaken yet for ovarian cancer. Furthermore, breast cancer in Russia and some neighboring countries is caused not only by mutations in BRCA genes, but also by heterozygous inactivation of the CHEK2 and NBS1 [42]. It remains unclear, whether the latter 2 genes contribute to OC predisposition as well, or, vice versa, their impact is limited by breast cancer risk.

## Materials and methods

Ovarian cancer cases were collected in 2 geographically distinct regions of Russian Federation. 290 patients (mean age: 53 years; age range: 21–89 years) were recruited in the N.N. Petrov Institute of Oncology (St.-Petersburg), which provides the treatment to the residents of North-Western Russia. In addition, the study included 64 women with OC (mean age: 59 years; age range: 33–78 years) from the regional oncological hospital of the city of Krasnodar, which is located in Southern Russia, more than two thousands kilometers aside from the first spot of patients collection. Almost all patients had Slavic ethnic origin. DNA isolation, design of PCR assays and other relevant technical information is described in detail in our earlier report [42].

## Results

The observed frequencies of founder mutations are presented in the Table 1. BRCA1 5382insC mutation was by far the most prevalent, accounting for 28/290 (9.7%) OC cases from the North-West and 11/64 (17.2%) from the South of Russia. Jewish BRCA1 variant, BRCA1 185delAG, was the second by prevalence: it was detected in 3/290 (1.0%) cases from St.-Petersburg and in 1/64 (1.6%) patients from Krasnodar. BRCA1 4153delA mutation was revealed only in 2/290 (0.7%) subjects from the first cohort, and BRCA1 300T>G was not observed in any sample tested. When both patients groups were pooled together, the occurrence of founder BRCA1 mutations approached to 12.7% (45/354). Other breast cancer associated founder mutations (BRCA2, CHEK2, NBS1) were detected only in single patients. Interestingly, 1 OC patient from Krasnodar carried both BRCA1 5382insC and NBS1 657del5 mutation; tumor tissue from this woman demonstrated loss of heterozygosity of the wild-type NBS1 allele, but intact status of BRCA1 gene. This unique case is presented in detail in a separate publication [43]. Based on the results of DNA test and the history of cancer diseases in her family, this patient opted for prophylactic subcutaneous nipple-sparing mastectomy coupled with the immediate breast reconstruction by silicone implant; morphological examination of the excised mammary tissue revealed no evidence for the tumor growth.

**Table 1:** Founder mutations in Russian ovarian cancer cases

	**St.-Petersburg (n = 290)**	**Krasnodar (n = 64)**
BRCA1 5382insC	28 (9.7%)	11 (17.2%)*

BRCA1 4153delA	2 (0.7%)	-

BRCA1 185delAG	3 (1.0%)	1 (1.6%)

BRCA1 300T>G	-	-

BRCA2 6174delT	1 (0.3%)	-

CHEK2 1100delC	2 (0.7%)	-

CHEK2 IVS2+1G>A	-	-

NBS1 657del5	-	1 (1.6%)*


Total carriers	36 (12.4%)	12 (18.8%)*

*1 patient from Krasnodar carried both BRCA1 5382insC and NBS1 657del5 mutations.

Distribution of founder mutations in various OC subgroups is analyzed in Table 2. Patients from St.-Petersburg did not demonstrate association with early age at onset, while the mutation carriers were somewhat overrepresented in younger patients from Krasnodar (10/34 (29.4%) in patients aged </= 60 years *versus *2/30 (6.7%) in older women; p = 0.02). Family history records were available only for the patients from the North-West; no association between the presence of the germ-line mutations and reporting OC or BC in a first-degree relative has been observed. Furthermore, there was no significant difference between carriers and non-carriers with respect to other clinico-pathological parameters of the disease.

**Table 2:** Frequencies of founder mutations in distinct categories of ovarian cancer patients

**Clinical variable**	**St.-Petersburg (n = 290)**	**Krasnodar (n = 64)**
Age at onset (years)		

< 41	2/36 (5.6%)	1/1 (100.0%)

41–60	22/172 (12.8%)	9/33 (27.2%)

> 60	12/82 (14.6%)	2/30 (6.7%)

Non-informative	-	


Family history*		

Positive	1/9 (11.1%)	-

Negative	33/266 (12.4%)	-

Non-informative	2/15 (13.3%)	12/64 (18.8%)


T status		

T1	2/52 (3.8%)	3/10 (30.0%)

T>1	32/220 (14.5%)	8/53 (15.1%)

Non-informative	2/18 (11.1%)	1/1 (100.0%)


N status		

N0	12/109 (11.0%)	1/4 (25.0%)

N1	7/53 (13.2%)	1/1 (100.0%)

N2	1/1 (100.0%)	-

Non-informative	16/127 (12.6%)	10/59 (16.9%)


M status		

M0	22/186 (11.8%)	11/58 (19.0%)

M1	6/63 (9.5%)	0/4 (0.0%)

Non-informative	8/41 (19.5%)	1/2 (50.0%)


Tumor grade		

1–2	7/96 (7.3%)	9/48 (18.8%)

3	23/154 (14.9%)	3/16 (18.8%)

Non-informative	6/40 (15.0%)	-


Tumor histology		

Serous adenocarcinoma	36/250 (14.4%)	7/41 (17.1%)

Mucinous adenocarcinoma	0/9 (0.0%)	0/2 (0.0%)

Adenocarcinoma, not otherwise specified	0/16 (0.0%)	3/11 (27.3%)

Other	0/15 (0.0%)	2/10 (20.0%)

*Family history was defined as the presence of breast and/or ovarian cancer in mother or sister.

## Discussion

This study was designed to analyze the impact of selected founder mutations in OC morbidity in Russia. The panel of genetic variants included several alleles, which are characteristic for Slavic people and/or residents of Baltic regions (BRCA1 5382insC, BRCA1 4153delA, BRCA1 300T>G, CHEK2 1100delC, CHEK2 IVS2+1G>A, NBS1 657del5), and/or European Jews (BRCA1 185delAG, BRCA2 6174delT). This study confirmed the utmost role of BRCA1 5382insC mutation in cancer morbidity: indeed, this single genetic defect is responsible for as many as 1 out of 9 ovarian cancers and 1 out of 25 breast cancers occurring in Russia. Furthermore, BRCA1 5382insC demonstrates unique geographic spread, being among the most prevalent BRCA variants in Poland, Byelorussia, Baltic republics, various cities of Russian Federation (St.-Petersburg, Tomsk, and Krasnodar), and possibly some Mediterranean countries [42,44-49]. The remarkable role of the founder effect contradicts to the wide-spread view that the extreme complexity of the history of Russian Empire resulted in huge genetic diversity of Russian residents. It is important to emphasize, that recent investigations show surprisingly high level of genetic homogeneity for the 3 analyzed Slavic-speaking populations, i.e. Russians, Ukrainians and Poles [50]. These unexpected data are in good agreement with the lack of language diversity within the Russian Federation: astonishingly, people residing nearby Baltic sea or in the Far East speak exactly the same dialect, despite being separated by the distance of 11000 kilometers.

Other BRCA1 founder mutations are relatively rare in Russian ovarian or breast cancer cases [42]. BRCA1 4153delA allele was initially considered to be characteristic for familial OC in Russia [44]; furthermore, it was suggested that this variant confers specific predisposition to the ovarian but not breast carcinogenesis [25,48]. Our data do not confirm these statements. Jewish BRCA1 185delAG allele is occasionally detected in Russian OC and BC cases, while Baltic BRCA1 300T>G variant demonstrates null occurrence and therefore has to be excluded from the local diagnostic panel [42]. Taken together with other reports on OC patients or healthy controls [36-38,51], this study does not support the role of CHEK2 gene lesions in OC predisposition (Table 1). On the other hand, the possibility of limited contribution of NBS1 germ-line mutations in OC risk cannot be fully excluded. Previously, Plisiecka-Hałasa et al. [39] identified 2 NBS1 657del5 mutation carriers among 117 OC patients. In the present study, the frequency of NBS1 heterozygosity in OC patients (0.3%) did not differ significantly from the estimate obtained in healthy Russian women (0.6%) [52]. However, tumor tissue from the BRCA1/NBS1 heterozygous carrier from our OC group contained biallelic inactivation of NBS1 but not BRCA1 [43].

Most of the published investigations reported a correlation between the presence of BRCA1 mutation and family history of breast/ovarian cancer as well as earlier onset of the disease. Some reports also noticed association of BRCA1 heterozygosity with non-mucinous tumor histology, high tumor grade, and improved survival (see additional file 1). Nevertheless, almost all investigators agree that neither lack of family history nor late disease onset are reliable indicators of BRCA1 wild-type status in OC patients, therefore DNA testing of non-selected ovarian cancer cases is recommended by some laboratories. Our study failed to detect relationships between BRCA1 germ-line mutations and first-degree family history. There are some factors, which could complicate the analysis of pedigrees for BC and OC cases. First of all, BRCA1-related cancers are mainly gender-specific: i.e. male carriers of BRCA1 defects do not experience significantly elevated cancer risk due to absence of the main target organs (breasts and ovaries); therefore, family history negative BRCA1-associated BC and OC are particularly likely in case of paternal transmission of the mutation. Other confounding effects are related to specific circumstances of recent history of Russian Federation. Huge human losses caused by historical turbulences in the XX century, taken together with limited average life expectancy, led to the situation when many BRCA1 carriers simply failed to achieve the age of cancer manifestation due to premature death. Another factor, that may increase the number of false-negative pedigrees, is a relatively low number of children per family in the modern Russia; in other words, many of questioned patients could recall only a small number if any of their adult female relatives. Finally, an analysis of heredity is frequently ignored by Russian medical professionals, i.e. family history records in medical charts may simply lack a sufficient level of accuracy.

## Conclusion

This study indicates that all ovarian cancer patients of Russian ethnic origin have to be screened for the presence of a few BRCA1 founder mutations (BRCA1 5382insC, BRCA1 185delAG, BRCA1 4153delA). Introduction of this cheap DNA test into routine medical practice may help to identify yet healthy relatives of OC patients, who carry the same genetic defect and thus will benefit from tight surveillance and/or prophylactic surgery. Furthermore, recent findings suggest that BRCA1-associated ovarian cancers may require distinct strategy for the disease treatment [53].

## Abbreviations

BC: breast cancer; OC: ovarian cancer

## Competing interests

The authors declare that they have no competing interests.

## Authors' contributions

ENS, APS, AGI, AVT, SYM, AFU, NVP and ENI contributed in the conception of the study. NYS, DNP, TVG, OAZ, OSY, AVT, NNT, GAS, OSL, NYK, DEM, SYM, AFU were responsible for data collection. ENS, APS, NVP, ENI performed data analysis. ENI wrote the manuscript. All authors approved the final version.

## Supplementary Material

Additional file 1 **Germ-line mutations in non-selected ovarian cancer patients.** Table-format summary of the literature data on BRCA1, BRCA2, CHEK2, and NBS1 germ-line mutations in non-selected ovarian cancer patients Click here for file
