# Supplementary material for: High frequency of BRCA1, but not CHEK2 or NBS1 (NBN), founder mutations in Russian ovarian cancer patients
Source: Hered Cancer Clin Pract. 2009 Feb 25;7(1):5. doi: 10.1186/1897-4287-7-5 (PMC2664323; doi:10.1186/1897-4287-7-5)
Supplement: Additional file 1 — Germ-line mutations in non-selected ovarian cancer patients. Table-format summary of the literature data on BRCA1, BRCA2, CHEK2, and NBS1 germ-line mutations in non-selected ovarian cancer patients [file 1897-4287-7-5-S1.doc]

Germ-line mutations in non-selected ovarian cancer patients

| **Country / ethnicity** | **Mutation** | **Frequency** | **Clinical correlations*** | **Reference** |
| --- | --- | --- | --- | --- |
| **BRCA1 and/or BRCA2** | | | | |
| Ashkenazi Jews (Israel) | BRCA1, BRCA2: founder | 13/21 (61.9%) | n.e. | 2 |
| Ashkenazi Jews (Israel) | BRCA1, BRCA2: founder | 17/29 (58.6%) | Family history | 3 |
| Ashkenazi Jews (Israel) | BRCA1, BRCA2: founder | 27/118 (22.9%) | Younger age and family history (for BRCA1 but not BRCA2), improved survival | 4 |
| Ashkenazi Jews (Canada) | BRCA1, BRCA2: founder | 34/71 (47.9%) | Younger age (for BRCA1 but not BRCA2), improved survival | 5 |
| Ashkenazi Jews (USA) | BRCA1, BRCA2: founder | 23/92 (25.0%) | Younger age (for BRCA1 but not BRCA2), family history; no association with tumor histology, stage, grade | 6 |
| Ashkenazi Jews (USA) | BRCA1, BRCA2: founder | 88/189 (46.6%) | Younger age (for BRCA1 but not BRCA2), longer disease-free interval, improved survival; no association with tumor histology, stage, grade | 7 |
| Ashkenazi Jews (USA) | BRCA1, BRCA2: founder | 147/382 (38.5%) | n.e. | 8 |
| Ashkenazi Jews (Israel, North America) | BRCA1, BRCA2 (founder) + sequencing analysis of selected regions of BRCA1 and BRCA2 in selected cases | 86/208 (41.3%) | Younger age (for BRCA1 but not BRCA2), family history, non-mucinous and non-clear-cell histology, high tumor grade; no association with stage | 9 |
| Austria | BRCA1: founder | 2/122 (1.6%) | n.a. | 10 |
| Canada (French) | BRCA1, BRCA2: founder | 8/99 (8.1%) | Younger age (for BRCA1 but not BRCA2), family history | 11 |
| Canada | BRCA1, BRCA2 | 60/515 (11.7%) | Younger age (for BRCA1 but not BRCA2), family history | 12 |
| Czechia | BRCA1 | 3/30 (10.0%) | n.e. | 13 |
| Finland | BRCA1, BRCA2: founder | 13/233 (5.6%) | Family history | 14 |
| Hungary | BRCA1, BRCA2: founder | 10/90 (11.1%) | No association with age, tumor histology, stage | 15 |
| Iceland | BRCA2: founder | 3/38 (7.9%) | n.e. | 16 |
| Israel | BRCA1, BRCA2: founder | 229/779 (29.4%) | Ashkenazi ancestry, younger age (for BRCA1 but not BRCA2), family history, non-mucinous histology, higher grade, advanced stage; improved survival [18, 19] | 17 |
| Japan | BRCA1 | 3/76 (3.9%) | n.e. | 20 |
| Korea | BRCA1, BRCA2 | 1/37 (2.7%) | n.a. | 21 |
| Norway | BRCA1: founder | 18/615 (2.9%) | Younger age, family history, advanced stage and grade | 22 |
| Netherlands | BRCA1, BRCA2 | 5/85 (5.9%) | n.e. | 23 |
| Pakistan | BRCA1, BRCA2 | 19/120 (15.8%) | Association with older age at diagnosis | 24 |
| Poland | BRCA1: founder | 49/364 (13.5%) | Younger age, family history | 25 |
| Poland | BRCA1 (founder) + sequencing analysis of selected regions of BRCA1 and BRCA2 in selected cases | 21/151 (13.9%) | Younger age (for BRCA1, but not BRCA2), improved survival; no association with stage, grade, histological type | 26 |
| Russia | BRCA1, BRCA2: founder | 46/354 (13.0%) | No association with first-degree family history; association with younger age st onset in patients from the South, but not from the North-West of Russia | This study |
| Sweden | BRCA1. BRCA2 | 13/161 (8.1%) | Family history | 27 |
| Turkey | BRCA1. BRCA2 | 17/102 (16.7%) | Younger age, family history (for BRCA1), serous histology | 28 |
| UK | BRCA1 | 13/355 (3.7%); study is limited by patients < 70 years old | n.e. | 29 |
| USA | BRCA1 | 7/115 (6.1%) | Family history | 30 |
| USA | BRCA2 | 4/130 (3.1%) | n.e. | 31 |
| USA | BRCA1, BRCA2 | 10/116 (8.6%) | Family history | 32 |
| USA | BRCA1 | 4/120 (3.3%) | n.e. | 33 |
| USA | BRCA1 | 12/258 (4.7%) | Younger age, family history | 34 |
| USA | BRCA1, BRCA2 | 32/209 (15.3%) | Younger age (for BRCA1, but not BRCA2), family history; non-mucinous histology | 35 |
| **CHEK2** | | | | |
| Poland | CHEK2: founder | 0/209 (0.0%) | n.a. | 36 |
| Russia | CHEK2: founder | 2/354 (0.6%) | n.a. | This study |
| USA | CHEK2: founder | 0/751 (0.0%) | n.a. | 37 |
| USA | CHEK2 | 0/38 (0.0%) | n.a. | 38 |
| **NBS1** | | | | |
| Poland | NBS1 | 2/117 (1.7%) | n.a. | 39 |
| Poland | NBS1: founder | 0/108 (0%) | n.a. | 40 |
| Russia | NBS1: founder | 1/354 (0.3%) | n.a. | This study |

* n.e. - not evaluated; n.a. – not applicable due to low number of mutation carriers
